# Three-Dimensional (3D) Bicontinuous Hierarchically Porous Mn_2_O_3_ Single Crystals for High Performance Lithium-Ion Batteries

**DOI:** 10.1038/srep14686

**Published:** 2015-10-06

**Authors:** Shao-Zhuan Huang, Jun Jin, Yi Cai, Yu Li, Zhao Deng, Jun-Yang Zeng, Jing Liu, Chao Wang, Tawfique Hasan, Bao-Lian Su

**Affiliations:** 1 Laboratory of Living Materials at the State Key Laboratory of Advanced Technology for Materials Synthesis and Processing, Wuhan University of Technology, 122 Luoshi Road, 430070, Wuhan, Hubei, China; 2 Cambridge Graphene Centre, University of Cambridge, Cambridge, CB3 0FA, United Kingdom; 3 Nanoscience Centre, University of Cambridge, Cambridge CB3 0FF, United Kingdom; 4 Laboratory of Inorganic Materials Chemistry (CMI), University of Namur, 61 rue de Bruxelles, B-5000 Namur, Belgium; 5 Department of Chemistry and Clare Hall, University of Cambridge, Cambridge, CB2 1EW, United Kingdom

## Abstract

Bicontinuous hierarchically porous Mn_2_O_3_ single crystals (BHP-Mn_2_O_3_-SCs) with uniform parallelepiped geometry and tunable sizes have been synthesized and used as anode materials for lithium-ion batteries (LIBs). The monodispersed BHP-Mn_2_O_3_-SCs exhibit high specific surface area and three dimensional interconnected bimodal mesoporosity throughout the entire crystal. Such hierarchical interpenetrating porous framework can not only provide a large number of active sites for Li ion insertion, but also good conductivity and short diffusion length for Li ions, leading to a high lithium storage capacity and enhanced rate capability. Furthermore, owing to their specific porosity, these BHP-Mn_2_O_3_-SCs as anode materials can accommodate the volume expansion/contraction that occurs with lithium insertion/extraction during discharge/charge processes, resulting in their good cycling performance. Our synthesized BHP-Mn_2_O_3_-SCs with a size of ~700 nm display the best electrochemical performance, with a large reversible capacity (845 mA h g^−1^ at 100 mA g^−1^ after 50 cycles), high coulombic efficiency (>95%), excellent cycling stability and superior rate capability (410 mA h g^−1^ at 1 Ag^−1^). These values are among the highest reported for Mn_2_O_3_-based bulk solids and nanostructures. Also, electrochemical impedance spectroscopy study demonstrates that the BHP-Mn_2_O_3_-SCs are suitable for charge transfer at the electrode/electrolyte interface.

Lithium-ion batteries (LIBs) are now found in a diverse range of applications, from advanced portable electronic devices and electric vehicles to smart grids. A wide variety of continually emerging new applications necessitates an evolution in LIB technology and the need to develop more stably performing batteries, with higher energy and power density. The key to achieving this requirement lies in the design of highly efficient battery electrode materials. Transition metal oxides have drawn considerable interest as potential anode materials for LIBs because of their higher specific capacities than graphite^1^,^2^,^3^. Recently, Mn_2_O_3_ has come into focus owing to its many potential advantages such as high theoretical capacity (1018 mA h g^−1^), low cost, significant thermal stability and lower operating voltage (average discharge voltage at 0.5 V and charge voltage at 1.2 V)^4^,^5^,^6^,^7^. However, a truly durable Mn_2_O_3_ electrode with high capacity and high-rate capability has not yet been achieved due to its large volume expansion and the collapse of its structure upon electrochemical cycling, leading to severe loss in capacity and poor cycle life^8^,^9^,^10^.

Porous micro/nanostructures have been demonstrated as ideal candidates to overcome the major limitations in developing high-performance LIBs. This is because such structures can enhance the electrochemical kinetics, shorten the diffusion distance for Lithium ions and accommodate the volume change during the cycling process^11^,^12^,^13^,^14^,^15^,^16^. In this regard, porous single crystals (SCs) can provide high accessibility for the electrolyte, allow efficient charge/discharge cycles of Li ions and offer higher crystallinity (and better conductivity) than porous polycrystalline materials^17^, thus improving the cycling stability and rate capability for LIBs. However, reports on porous Mn_2_O_3_ SCs for LIBs are very limited. In addition, the introduction of bicontinuous hierarchically porous structures in Mn_2_O_3_ SCs could further enhance the electrochemical performance of LIBs because this structure can provide more active sites for the lithiation/delithiation reaction and facilitate Li ion transportation^18^. Furthermore, developing a simple and reliable method for the fabrication of such hierarchically porous Mn_2_O_3_ SCs remains a great challenge.

Here we report synthesis of three-dimensional bicontinuous hierarchically porous Mn_2_O_3_ SCs (BHP-Mn_2_O_3_-SCs) with uniform parallelepiped geometry. The electrochemical properties show that the BHP-Mn_2_O_3_-SCs deliver an excellent reversible capacity even at high current density and exceptional rate capability with high specific capacity. Such high lithium storage capacity and rate capability are among the highest values obtained for BHP-Mn_2_O_3_-SCs. We attribute these to the bicontinuous interpenetrating framework of the Mn_2_O_3_-SCs with bimodal mesoporous structure, offering higher conductivities for charge transfer, large specific surface areas, short transport distances for the lithium-ion insertion reaction at the interface and freedom for volume change during the charge/discharge cycles.

## Results

**Figure 1:**
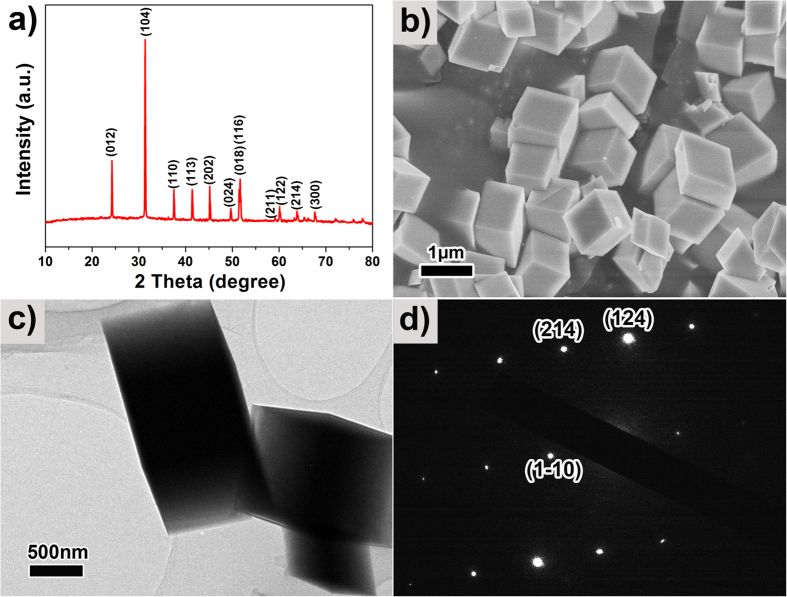
(**a**) XRD patterns; (**b**) SEM image; (**c**) TEM image; and (**d**) SAED pattern of the as-prepared MnCO_3_-15.

Figure 1a,b show the XRD pattern and SEM image of typical MnCO_3_ crystal synthesized using 15 mL of distilled-water (MnCO_3_-15), respectively. These crystals are precursor materials for the BHP-Mn_2_O_3_-SCs. All the peaks (Fig. 1a) can be indexed to MnCO_3_ crystalline phase in a rhombohedral lattice with space group *R*3*c* (JCPDS No: 44–1472). SEM image of this sample (Fig. 1b) reveals its monodispersity and uniform parallelepiped morphology with a size of ~700 nm. A typical TEM image and Selected Area Electron Diffraction (SAED) pattern of the MnCO_3_-15 sample are presented in Fig. 1c,d. The SAED patterns exhibit sharp diffraction spots, indicating the single crystal nature of MnCO_3_. Thermogravimetric (TG) analysis of the MnCO_3_-15 was carried out in air from room temperature to 900 °C, with a temperature ramping rate of 5 °C min^−1^ (see Supplementary Figure S1). The TG profile demonstrates that the decomposition starts at ~300 °C and terminates at ~500 °C, indicating 550 °C is an appropriate annealing temperature to obtain Mn_2_O_3_.

**Figure 2:**
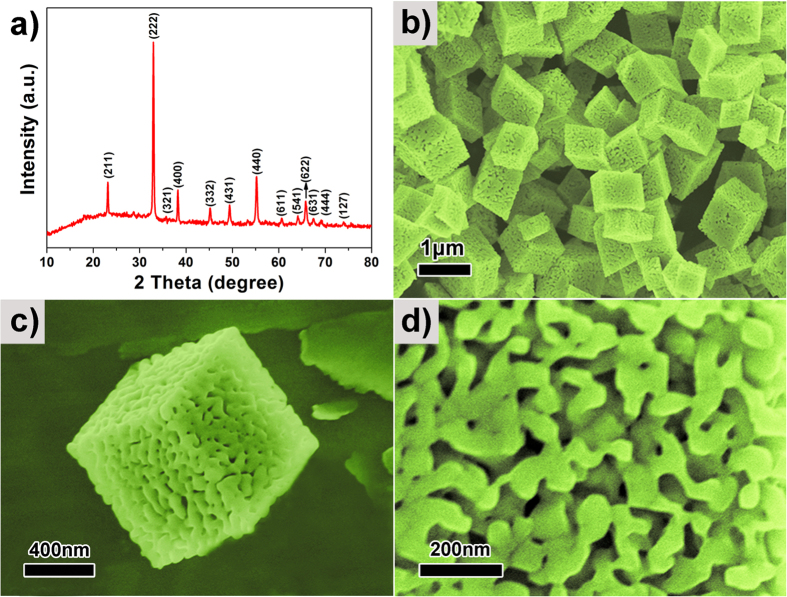
(**a**) XRD pattern; (**b–d**) SEM images of the BHP-Mn_2_O_3_-SCs (MO-15 sample).

Figure 2a shows the XRD pattern of the MO-15 sample produced from annealing MnCO_3_-15 crystals at 550 °C. This can be indexed to cubic Mn_2_O_3_ with a space group of *I*_*a3*_(JCPDS No: 71–0636). No impurities were detected. The sharp, intense XRD peaks indicate that the sample is highly crystalline. Typical SEM images of sample MO-15 are displayed in Fig. 2b–d. The low magnification SEM images (Fig. 2b,c) show that the MO-15 sample has a highly porous parallelepiped geometry with excellent uniformity and marginally reduced crystal size after thermal decomposition of MnCO_3_ in air accompanied by release of CO_2_. High magnification SEM image (Fig. 2d) reveals that the MO-15 sample exhibits a bicontinuous and interpenetrating framework^19^,^20^, consisting of quasi-periodic interconnected bimodal mesoporous channels.

**Figure 3:**
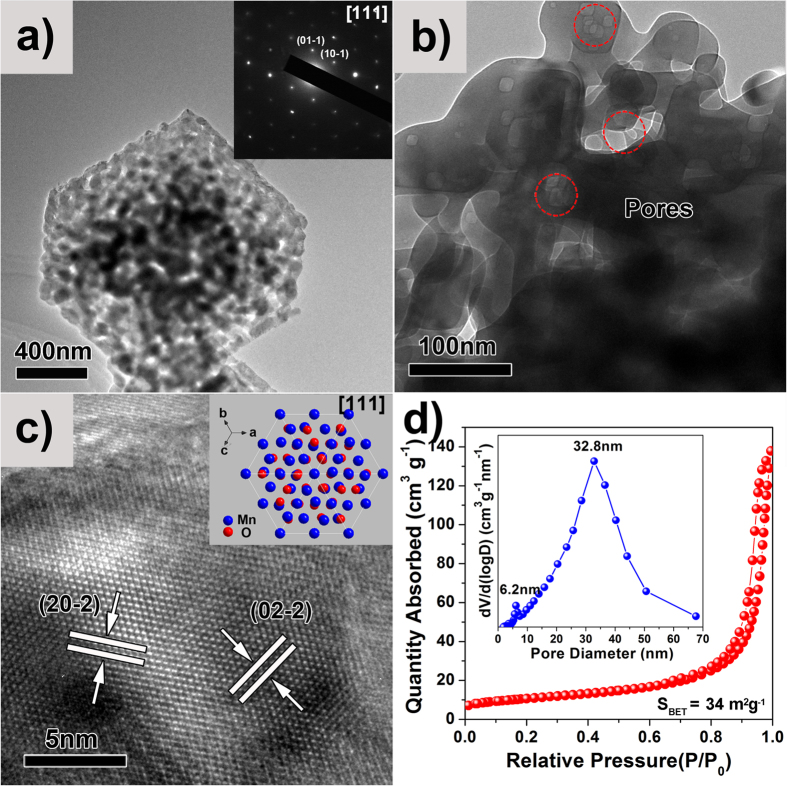
TEM images and N_2_ sorption isotherm of the BHP-Mn_2_O_3_-SCs (MO-15 sample): (**a**) low magnification TEM image, the inset in (**a**) is the corresponding SAED pattern; (**b**) high magnification TEM image; (**c**) HRTEM image, the inset in (**c**) is the crystal structure of Mn_2_O_3_ along the [111] direction; (**d**) N_2_ sorption isotherm, the inset in (**d**) is the pore size distribution.

Figure 3 displays the TEM images and N_2_ sorption isotherm of MO-15. Low magnification TEM image shows an average size of ~700 nm (see Supplementary Figure S2), consistent with the SEM images shown in Fig. 2b,c. Figure 3a presents a typical TEM image of an individual Mn_2_O_3_ parallelepiped single crystal, revealing that MO-15 exhibits a bicontinuous hierarchical structure with interconnected porous channels. The SAED patterns (Fig. 3a, inset) of one Mn_2_O_3_ parallelepiped single crystal demonstrate marginally sharper diffraction spots, clearly indicating the single crystal nature of Mn_2_O_3_ with ordered network arranged along the [111] direction. A higher magnification TEM image (Fig. 3b) further reveals that the Mn_2_O_3_ parallelepiped single crystals contain a bicontinuous structure with interconnected hierarchical bimodal mesoporosities (the corresponding enlarged TEM image is shown in Supplementary Figure S3). The HRTEM image in Fig. 3c demonstrates well-resolved lattice fringes with an interplanar spacing of 0.333 nm, corresponding to the (20-2) plane and (02–2) plane along the [111] direction. The inset in Fig. 3c shows the atomic arrangement of the cubic Mn_2_O_3_ along the [111] direction. N_2_ adsorption-desorption isotherm of the MO-15 in Fig. 3d shows a typical Type IV isotherm with H1 type hysteresis, revealing a uniform mesoporous structure^21^. The BET specific surface area is 34 m^2^ g^−1^. The pore size distribution plot (inset of Fig. 3d) shows a large-pore-size distribution of ~32.8 nm and another small-pore-size distribution of ~6.2 nm, confirming the SEM and TEM observations of bimodal mesoporosity of these Mn_2_O_3_ SCs.

**Figure 4:**
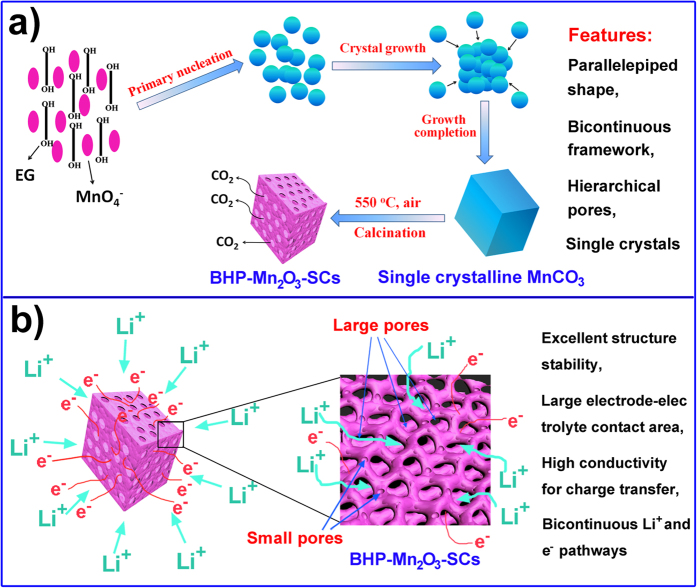
(**a**) Illustration of the preparation processes of BHP-Mn_2_O_3_-SCs; (**b**) illustration of lithium insertion mechanism in the BHP-Mn_2_O_3_-SCs with fast electron transportation, large electrode-electrolyte contact area and shortened Li ion transport pathways.

Based on the SEM and TEM observations, a formation mechanism of the BHP-Mn_2_O_3_-SCs is illustrated in Fig. 4a. First, the MnO_4_^−^ ions assemble together with the functional Ethylene Glycol (EG) molecules under stirring, followed by the formation of primary nanoparticles during redox reactions between the MnO_4_^−^ ions and hydroxyl (-OH) groups of EG. Subsequently, the primary nanoparticles rapidly grow along a particular crystallographic orientation, to form MnCO_3_ SCs via the Ostwald ripening mechanism^22^. After 0.5 h reaction, parallelepiped shaped MnCO_3_ crystals form (see Supplementary Figure S4), indicating very fast growth of the crystals during the reaction. Finally, the porous Mn_2_O_3_ SCs are obtained by thermal decomposition of MnCO_3_ under air atmosphere. The reaction equations can be written as:


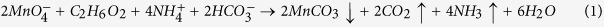



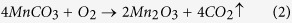


During the calcination process, the hierarchical porosities are formed by thermal decomposition of MnCO_3_, accompanied by gentle release of CO_2_. The release of CO_2_ from MnCO_3_ crystals induces a contraction of the crystalline structure to form a solid network by connection of the nanosubunits as nodes (Fig. 4b), resulting in a three dimensional hierarchically porous single crystals with interconnected bicontinuous bimodal mesoporous framework. A similar phase transformation has also been reported elsewhere^23^.

**Figure 5:**
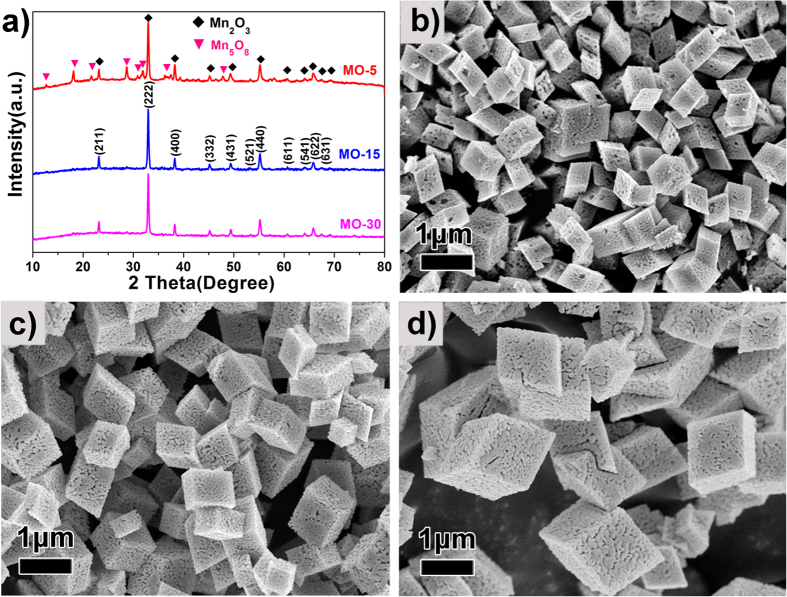
The XRD patterns and SEM images of the as-prepared MO-5, MO-15 and MO-30 samples: (**a**) XRD patterns; (**b**) MO-5; (**c**) MO-15; (**d**) MO-30.

The size of the MnCO_3_ SCs can further be tailored via controlling the amount of water in our reaction system^24^. By using 5 ml, 15 ml or 30 ml of water, three different sized MnCO_3_ SCs (MnCO_3_-5, MnCO_3_-15 and MnCO_3_-30) with 500 nm, 700 nm and 1.2 μm dimensions are obtained, respectively. Their XRD patterns and SEM images are presented in Figure S5 (see Supplementary). After calcination, MnCO_3_ crystals are transformed into three different sized Mn_2_O_3_ crystals (MO-5, MO-15 and MO-30, respectively). The corresponding XRD patterns and SEM images are shown in Fig. 5. Unlike MO-15 and MO-30, a competing Mn_5_O_8_ phase (JCPDS No: 39–1218) can be observed in the MO-5 sample, though the amount is minimal. This impurity could imply that the primary nanoparticles grow slower in the highly viscous solution compared with the nanoparticles in the low viscous solution^25^, leading to formation of MnCO_3_ and trace amount of other manganese compounds. The SEM images indicate that all the samples possess a hierarchially bicontinuous porous structure. This is confirmed by N_2_ adsorption-desorption (see Supplementary Figure S6). The isotherms of MO-5 and MO-30 are also of type IV with H1 type hysterisis with surface areas of 21 and 28 m^2^g^−1^, respectively. Both samples present two maximum in their pore size distribution curves at 33/47.1 nm and 26.1/37 nm, respectively (see Supplementary Figure S6a and S6b insets), confirming all the samples have a porous framework with bimodal mesoporosity. Figure S7 (see Supplementary) depicts the morphology and pore structure of a parallelepiped single crystal of MO-30, again confirming their bicontinuous hierarchical porous structural nature with its interconnected bimodal mesoporosities. The crystal size and hierarchical porous structure can thus be easily adjusted during the formation of MnCO_3_ SCs.

**Figure 6:**
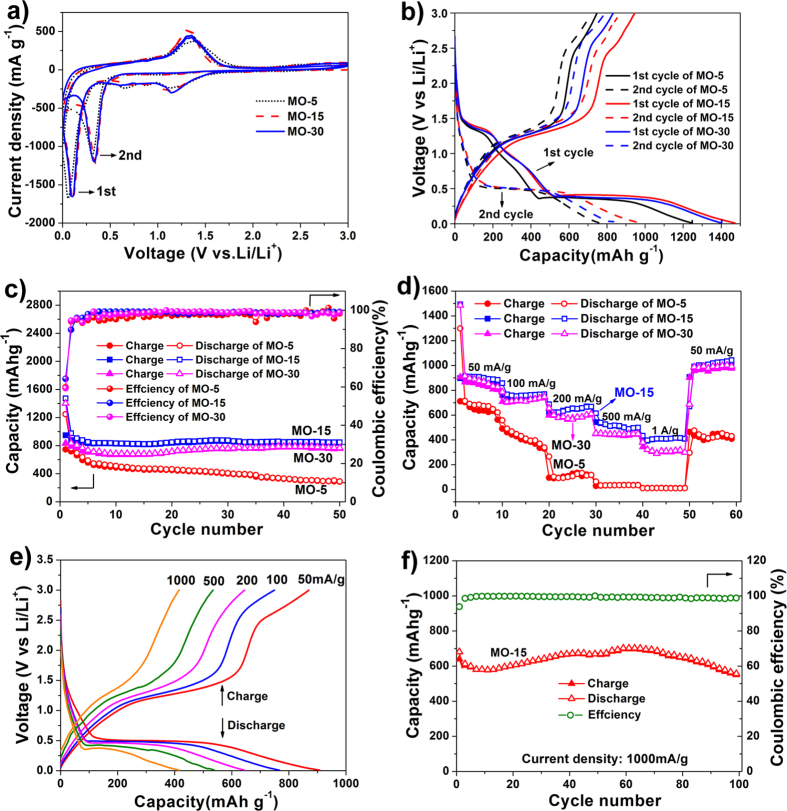
Electrochemical performances of the MO-5, MO-15 and MO-30 electrodes. (**a**) Cyclic voltammograms at a scanning rate of 0.1 mV s^−1^ in the voltage range of 0 ~ 3 V versus Li/Li^+^, respectively; (**b**) the first and second charge-discharge profiles at 100 mA g^−1^ in the voltage range of 0.01 V–3 V; (**c**) cycling performance and coulombic effciencies at 100 mA g^−1^; (**d**) charge-discharge capacities at various rates; (**e**) charge-discharge profiles of MO-15 at various rates; (**f**) cycling performance of MO-15 at 1000 mA g^−1^ following the rate test.

The electrochemical behaviours of MO-5, MO-15 and MO-30 are studied by cyclic voltammetry (CV) for the first and second cycles (Fig. 6a) in the voltage range of 3-0 V versus Li/Li^+^ at a scan rate of 0.1 mV s^−1^ (see Supplementary Figure S8 for the individual plots). For the first cycle, both the MO-15 and MO-30 samples deliver three cathodic peaks. The two broad peaks located at 0.68 V and 1.2 V are attributed to the decomposition of the electrolyte solvent and the formation of the solid electrolyte interphase (SEI) layer, as well as the reduction of Mn^3+^ to Mn^2+^ 26,27,28,29. Another distinct peak at ~0.1 V is ascribed to a further reduction of MnO to Mn^30^,^31^,^32^. Compared with MO-15 and MO-30, MO-5 exhibits an additional cathodic peak at ~0.82 V. This could be attributed to the electrochemical reaction between the Mn_5_O_8_ impurity and Li ions. In the anodic scan, an anodic peak at ~1.3 V can be observed for all three samples. This peak is associated with the oxidation of Mn to MnO. Compared with the first cathodic process, the peak current density and integrated area of the second cathodic process are smaller, indicating the initial discharge capacity decays after the first charging process.

Figure 6b shows the first and second discharge/charge profiles of the MO-5, MO-15 and MO-30 at a current density of 100 mA g^−1^. Consistent with the two reduction peaks in CV curves, one inclined voltage plateau at ~1.3 V and one small inflection between 1.0-0.5 V can be observed for all the samples in the first discharge. These are ascribed to the reduction of Mn^3+^ to Mn^2+^ and the formation of a SEI layer, respectively^33^,^34^,^35^,^36^. The wide voltage plateau is observed at ~0.38 V, corresponding to the complete reduction of MnO to Mn and the formation of amorphous Li_2_O^31^,^37^. In the first charge, there is a well-defined voltage plateau ~1.3 V. This matches well with the anodic peak centered at 1.3 V in the CV curves and is associated with the reversible oxidation of Mn^0^ to Mn^2+^. It is worth noting that an additional shoulder peak ~2.3 V can be observed. This may correspond to the further oxidation of MnO to MnO_x_ (1.0 ≤ x < 1.5), since it cannot be observed in the charge curve of MnO^38^.

Figure 6c depicts the cycling performance of the MO-5, MO-15 and MO-30 samples at a current density of 100 mA g^−1^. All the samples exhibit very high initial discharge capacities (1248 mA h g^−1^, 1473 mA h g^−1^ and 1403 mA h g^−1^ for the MO-5, MO-15 and MO-30 samples, respectively) in direct relation with the high surface areas of the three samples. The reduced second discharge capacities (763 mA h g^−1^, 975 mA h g^−1^ and 835 mA h g^−1^ for the MO-5, MO-15 and MO-30 samples, respectively) are consistent with the capacity values shown in Fig. 6b. The initial capacity loss can be attributed to Li consumption associated with the SEI formation^39^. After 50 cycles, the specific capacities of the MO-15 and MO-30 samples can be maintained at levels as high as 845 mA h g^−1^ and 765 mA h g^−1^, respectively, revealing their great cycling stability and reversibility as anode materials for LIBs. More interestingly, the MO-15 sample delivers a higher specific capacity than the MO-30 sample, which may be ascribed to the higher BET specific surface area of the MO-15 sample (34 m^2^ g^−1^) than that of the MO-30 sample (28 m^2^ g^−1^). However, the MO-5 sample shows a much poorer cycling performance (only 300 mA h g^−1^ after 50 cycles, with 38.3% capacity retention). This is likely due to the Mn_5_O_8_ impurity blocking the surface pores of the MO-5 sample, resulting in its lower surface area (21 m^2^ g^−1^), and thus greatly impacting on the cycling stability of porous Mn_2_O_3_. It is worth noting that the capacities of the MO-15 and MO-30 samples are increased at first before reaching a steady level during the cycling process. This can be attributed to the reversible growth of pseudo-capacitive polymeric gel-like film as well as gradual SEI formation^40^,^41^,^42^. Additionally, the MO-15 sample delivers the highest initial coulombic efficiency of about 64.3% and then remains steady at >95% after the second cycle.

Rate performances of the MO-5, MO-15 and MO-30 samples are also evaluated (Fig. 6d,e). With the benefits of a three dimensional bicontinuous hierarchically porous SC framework with bimodal pore distribution, all the samples exhibit an excellent cycling response to a continuously varying current rate. In consistence with the tendency in Fig. 6c, the rate performances of the MO-15 and MO-30 samples are significantly better than that of the MO-5 sample. The poorer performance of the MO-5 sample could again be attributed to the presence of the Mn_5_O_8_ impurity, which may significantly affect the stability of the electrode as well as the rate performance. At current densities of 50, 100, 200, 500 and 1000 mA g^−1^, the reversible capacities of the MO-15 sample are around ~910, 766, 660, 534 and 416 mA h g^−1^, respectively. When the current density is reduced back to 50 mA g^−1^, the specific capacity of the MO-15 sample (1016 mA h g^−1^) becomes even higher than that of the initial performance at 50 mA g^−1^ (890 mA h g^−1^). This is ascribed to the reversible growth of pseudo-capacitive polymeric gel-like film as well as gradual SEI formation^40^,^41^,^42^. These results imply that the BHP-Mn_2_O_3_-SCs are robust and very effective for high rate application in LIBs. It is of great interest to observe that when the current density is set to 1 A g^−1^ again using the same button battery following the rate capability test, specific capacity of 600 mA h g^−1^ with ~100% coulombic efficiency can be attained for the electrode. This is much higher than the capacity shown in Fig. 6d (416 mA h g^−1^) at the same current density and is due to the reversible growth of pseudocapacitive polymeric gel-like film and gradual SEI formation as mentioned above (Fig. 6f).

**Figure 7:**
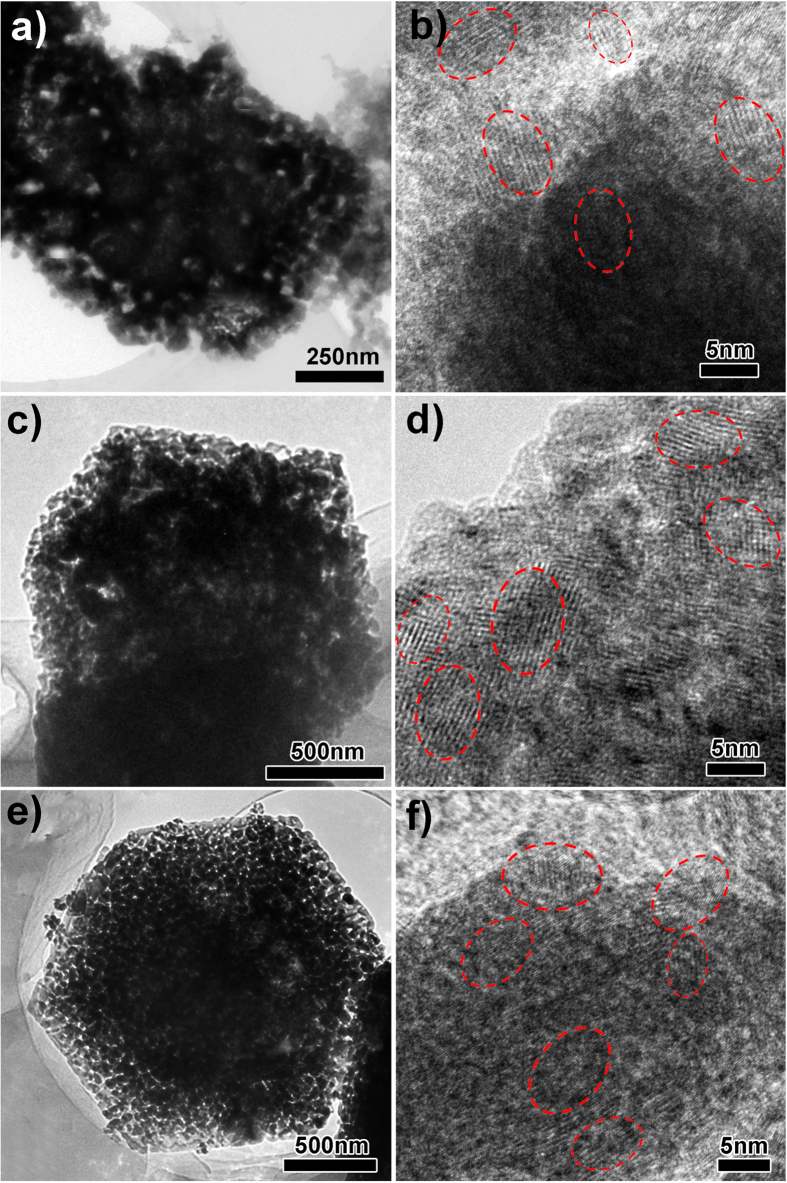
The *ex-situ* TEM and HRTEM characterizations of MO-5, MO-15 and MO-30 electrodes after 50 discharge-charge cycles at 100 mA g^−1^: (**a–b**) MO-15; (**c–d**) MO-15 and (**e–f**) MO-30.

The high lithium storage capacity and excellent rate capability of MO-15 and MO-30 samples can be ascribed to the three dimensional interconnected porous framework that provides excellent structural stability, bicontinuous Li^+^ and e^−^ pathways, and good electronic conductivity. To further understand the Li^+^ storage property and structural stability of the BHP-Mn_2_O_3_-SCs, post-mortem investigations after 50 discharge-charge cycles at 100 mA g^−1^ are carried out by SEM, TEM and HRTEM (see Supplementary Figure S9 and Fig. 7). We find that after 50 cycles, the morphology and porous structure of MO-15 and MO-30 are perfectly preserved without any structural alterations, while the structure of MO-5 is slightly destroyed, which partially accounts for the capacity decay of MO-5 (Fig. 7a). In addition, HRTEM images (Fig. 7b,d,f) reveal that after 50 cycles, the Mn_2_O_3_ electrodes are changed to polycrystalline structure. Although partial amorphization happens on the Mn_2_O_3_ electrodes, they still show high crystallinity, which ensures good conductivity of the electrodes upon cycling. Furthermore, to verify the structure superiority imposed on electrochemical behavior, we compare the electrochemical performance of BHP-Mn_2_O_3_-SCs with the solid Mn_2_O_3_ nanoparticles (see Supplementary Figures S10a and b). The electrochemical results distinctly demonstrate the MO-15 and MO-30 samples show better cycling performance and rate capability than those of Mn_2_O_3_ spheres (see Supplementary Figures S10c and d), indicating the three dimensional interconnected porous framework is very favourable for the improvement of lithium storage performance.

**Figure 8:**
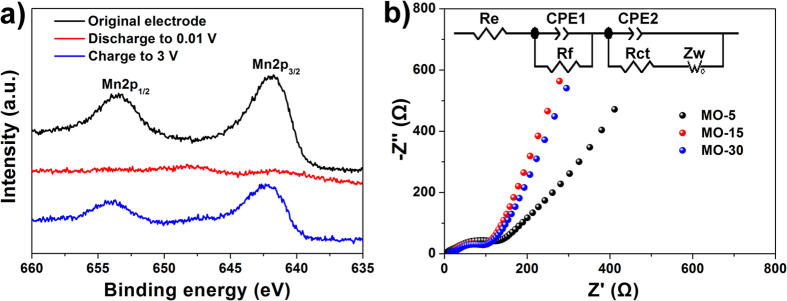
(**a**) XPS spectra of Mn 2p of the original MO-15 and discharge-charged MO-15 electrodes, respectively; (**b**) electrochemical impedance spectra of the electrodes of the MO-5, MO-15 and MO-30 electrodes after 10 cylces. The inset is the corresponding circuit diagram according to the EIS results.

*Ex-situ* XPS has been used to investigate the chemical states of the MO-15 electrode at different discharge/charge stages, as shown in Fig. 8a. As for the original electrode, the peaks of Mn 2p3/2 and Mn 2p1/2 are centered at 641.75 and 653.45 eV, respectively, and the spin-orbit splitting is 11.7 eV, matching well with the reported data of Mn_2_O_3_^43^,^44^. When the electrode is discharged to 0.01 V, the Mn 2p spectra nearly disappear, suggesting the metallic Mn materials are almost surrounded by the thickened SEI layer^45^. When the electrode is again charged to 3 V, the peaks of Mn 2p3/2 and Mn 2p1/2 shift to slightly higher energies, located at 642.35 and 653.70 eV, respectively. It is interesting to note that the spin-orbit splitting of the BHP-Mn_2_O_3_-SCs electrode (11.35 eV) is between those of Mn_2_O_3_ (11.7 eV)^44^ and MnO (11.2 eV)^46^,^47^, indicating our electrode is mainly composed of MnO_x_ (1.0 ≤ x < 1.5), which is consistent with the analysis in Fig. 6b.

To further investigate the excellent rate capability of the MO-15 and MO-30 samples, electrochemical impedance spectroscopy (EIS) measurements over the frequency range from 10 mHz to 100 kHz were carried out after the 10^th^ cycle at 100 mA g^−1^ current density. Figure 8b demonstrates that the diameters of the semicircle of the MO-15 and MO-30 samples are smaller than that of the MO-5 sample, indicating better electronic conductivity of the MO-15 and MO-30 samples. This confirms that the presence of Mn_5_O_8_ can not only affect the surface area but also the conductivity of Mn_2_O_3_. Therefore, with a combination of the highly reversible capacity, excellent cycling and high-rate performance, it can be concluded that the pure BHP-Mn_2_O_3_-SCs are a promising anode material candidate for high-performance LIBs.

To the best of our knowledge, such high lithium storage capacity and excellent rate capability are among the highest values obtained for the power performance of BHP-Mn_2_O_3_-SCs^48^, and are much superior to those of the previously reported Mn_2_O_3_ bulk solids^9^, Mn_2_O_3_ nanoparticles^10^, one-dimensional Mn_2_O_3_ materials^49^, porous Mn_2_O_3_ microspheres^11^, porous Mn_2_O_3_ nanoplates^32^ and hollow Mn_2_O_3_ microspheres^33^. This is due to the 3D bicontinuous hierarchical bimodal mesoporous network in Mn_2_O_3_ SCs we present here. The structure can accommodate large volume changes during the charge/discharge process and guarantees excellent structural stability, resulting in good cycling stability. In addition, the high crystallinity of Mn_2_O_3_ SCs ensures good conductivity for charge transfer. Also, the bicontinuous interpenetrating framework offers enhanced electron/ion transport pathways. And finally, the large specific surface area (34 m^2^ g^−1^) and hierarchically interconnected pores increase electrode-electrolyte contact, promoting high lithium storage capacity and superior rate capability.

## Conclusion

BHP-Mn_2_O_3_-SCs with uniform parallelepiped geometry have been successfully synthesized via the thermal decomposition of MnCO_3_ SCs. The BHP-Mn_2_O_3_-SCs exhibit a high specific surface area and three dimensional interconnected bicontinuous porous system with bimodal mesoporosities throughout the entire crystal. Three differently sized Mn_2_O_3_ SCs (500 nm, 700 nm and 1.2 μm) are prepared to investigate the relationship between the pore structure or crystal structure and the electrochemical performance. The results show that BHP-Mn_2_O_3_-SCs with a size ~700 nm display superior electrochemical performances with a large reversible capacity (845 mA h g^−1^ at 100 mA g^−1^ after 50 cycles), high coulombic efficiency (over 95% after the second cycle), excellent cycling stability and good rate capability (410 mA h g^−1^ at a current density of 1 A g^−1^), which can be attributed to the special hierarchically porous structure of the Mn_2_O_3_ SCs with high crystallinity and high porosity. Our BHP-Mn_2_O_3_-SC production strategy provides a useful approach for the design and synthesis of special morphologies of metal oxide SCs.

## Methods

### Synthesis of BHP-Mn_2_O_3_-SCs

In a typical synthesis, 0.474 g of KMnO_4_ was dispersed in 35 mL ethylene glycol (EG) and then stirred for 20 min. Subsequently, 1.2 g of NH_4_HCO_3_ dissolved in 15 mL distilled water was added into the above brown-black solution. After stirring for another 20 min, the solution was transferred into a thermostatic oil bath and heated to 80 °C with vigorous stirring for 9 h. The white precipitate obtained was repeatedly washed with ethanol and water until the organic reagents were removed. The obtained precipitate was then dried in air at 60 °C for 6 h. Finally, BHP-Mn_2_O_3_-SCs were obtained after annealing the white precipitate at 550 °C in air for 8 h and were designated as MO-15. For comparison, 5 mL and 30 mL distilled water were also used to prepare different sized BHP-Mn_2_O_3_-SCs, designated as MO-5 and MO-30, respectively.

For the synthesis of Mn_2_O_3_ nanoparticles: 0.474 g of KMnO_4_, 1.2 g of NH_4_HCO_3_ and 0.1 g of polyvinylpyrrolidone (PVP) were dispersed in 35 mL distilled water and stirred for 20 min. Subsequently, 70 mL isopropanol was added into the solution and stirred for another 20 min. The purple black solution was then transferred into a water bath and heated to 80 °C with vigorous stirring for 5 h. The obtained brown precipitate was washed with distilled water and ethanol repeatedly and placed into an oven at 60 °C for 4 h. Finally, the brown powder was annealed in air at 600 °C for 4 h to obtain the Mn_2_O_3_ nanoparticles.

### Materials characterization

The powder XRD patterns were obtained using a Bruker diffractometer at 40 kV, 40 mA, with Cu Kα_1_ radiation. The morphology of all the products was revealed using field emission scanning electron microscopy (FESEM, Hitachi S-4800). Transmission electron microscopy (TEM) was performed on a JEOL JEM-2100F with an acceleration voltage of 200 kV. The The N_2_ adsorption-desorption isotherms were measured at 77 K using a Micrometrics Tri Star II 3020 apparatus. Thermogravimetric (TG) analysis was performed using a simultaneous thermal analysis instrument (Setaram Labsys Evo S60/58458) at a temperature ramping rate of 5 °C min^−1^ in air. The surface electronic states of Mn were analyzed by X-ray photoelectron spectroscopy (XPS, VG Multilab 2000).

### Electrochemical measurements

The working electrodes were fabricated by using the BHP-Mn_2_O_3_-SCs as the active materials, conductive carbon blacks (Super-P) and polyvinylidene fluoride (PVDF) binder in a weight ratio of 70: 20: 10. The slurry was coated on a copper foil and dried in a vacuum oven at 120 °C for 12 h. Then the copper foil was cut into round flakelets with diameter of 8 mm. The average active material load of BHP-Mn_2_O_3_-SCs is ∼1.3 g. Electrochemical measurements were carried out via a CR2025 coin type cell using lithium pellets as the counter electrode and the reference electrode and a 1 M solution of LiPF_6_ in ethylene carbon (EC)/dimethyl carbonate (DMC) (1:1 w/w) as the electrolyte. The cells were assembled in an argon-filled glove-box. Cyclic Voltammetry (CV) measurements were carried out using a CHI 660D electrochemical workstation at a scanning rate of 0.1 mV s^−1^. Galvanostatic charge/discharge cycling was studied in a potential range of 0.01 V–3 V vs Li/Li^+^ with a multichannel battery testing system (LAND CT2001A). Electrochemical impedance spectra (EIS) were measured with an electrochemical workstation (Autolab PGSTAT 302N) in the frequency range 10 mHz to 100 kHz.

## Additional Information

**How to cite this article**: Huang, S.-Z. *et al.* Three-Dimensional (3D) Bicontinuous Hierarchically Porous Mn_2_O_3_ Single Crystals for High Performance Lithium-Ion Batteries. *Sci. Rep.* **5**, 14686; doi: 10.1038/srep14686 (2015).

## Supplementary Material

Supplementary Information
