# Supplementary material for: Three-Dimensional (3D) Bicontinuous Hierarchically Porous Mn2O3 Single Crystals for High Performance Lithium-Ion Batteries
Source: Sci Rep. 2015 Oct 6;5:14686. doi: 10.1038/srep14686 (PMC4593967; doi:10.1038/srep14686)

**Supplementary Information**

Three-Dimensional (3D) Bicontinuous Hierarchically Porous Mn_2_O_3_ Single Crystals for High Performance Lithium-Ion Batteries

Shao-Zhuan Huang^1^, Jun Jin^1^, Yi Cai^1^, Yu Li*^1^, Zhao Deng^1^, Jun-Yang Zeng^1^, Jing Liu^1^, Chao Wang^1^, Tawfique Hasan^2, 3^ and Bao-Lian Su*^1, 4, 5^

^1^Laboratory of Living Materials at the State Key Laboratory of Advanced Technology for Materials Synthesis and Processing, Wuhan University of Technology, 122 Luoshi Road, 430070, Wuhan, Hubei, China

^2^Cambridge Graphene Centre, University of Cambridge, Cambridge, CB3 0FA, United Kingdom

^3^Nanoscience Centre, University of Cambridge, Cambridge CB3 0FF, United Kingdom

^4^Laboratory of Inorganic Materials Chemistry (CMI), University of Namur, 61 rue de Bruxelles, B-5000 Namur, Belgium

^5^Department of Chemistry and Clare Hall, University of Cambridge, Cambridge, CB2 1EW, United Kingdom

Correspondence and requests for materials should be addressed to Y. L. ([yu.li@whut.edu.cn](mailto:yu.li@whut.edu.cn)). Tel: (+86) 27 87855322. Fax: (+86) 27 87879468 or B.L.S. ([bao-lian.su@unamur.be](mailto:bao-lian.su@unamur.be)). Tel: (+32) 81 724531. Fax: (+32) 81 725414.

**Figure S1.** TGA curves of the as-prepared MnCO_3_-15. It reveals two primary weight-loss stages. The first weight loss of ~1.1 wt% can be attributed to the desorption of physically adsorbed water on the sample while the second weight loss (from 300 °C to 520 °C) corresponds to the release of CO_2_ during thermal decomposition of MnCo_3_ to Mn_2_O_3_. (equation (2) in the main text). The mass loss with the release of CO_2_ is ~30 wt%, agreeing well with the theoretical value (31 wt%). After 520 °C, there is no mass change, indicating that 550 °C is a suitable calcination temperature to obtain pure Mn_2_O_3_.


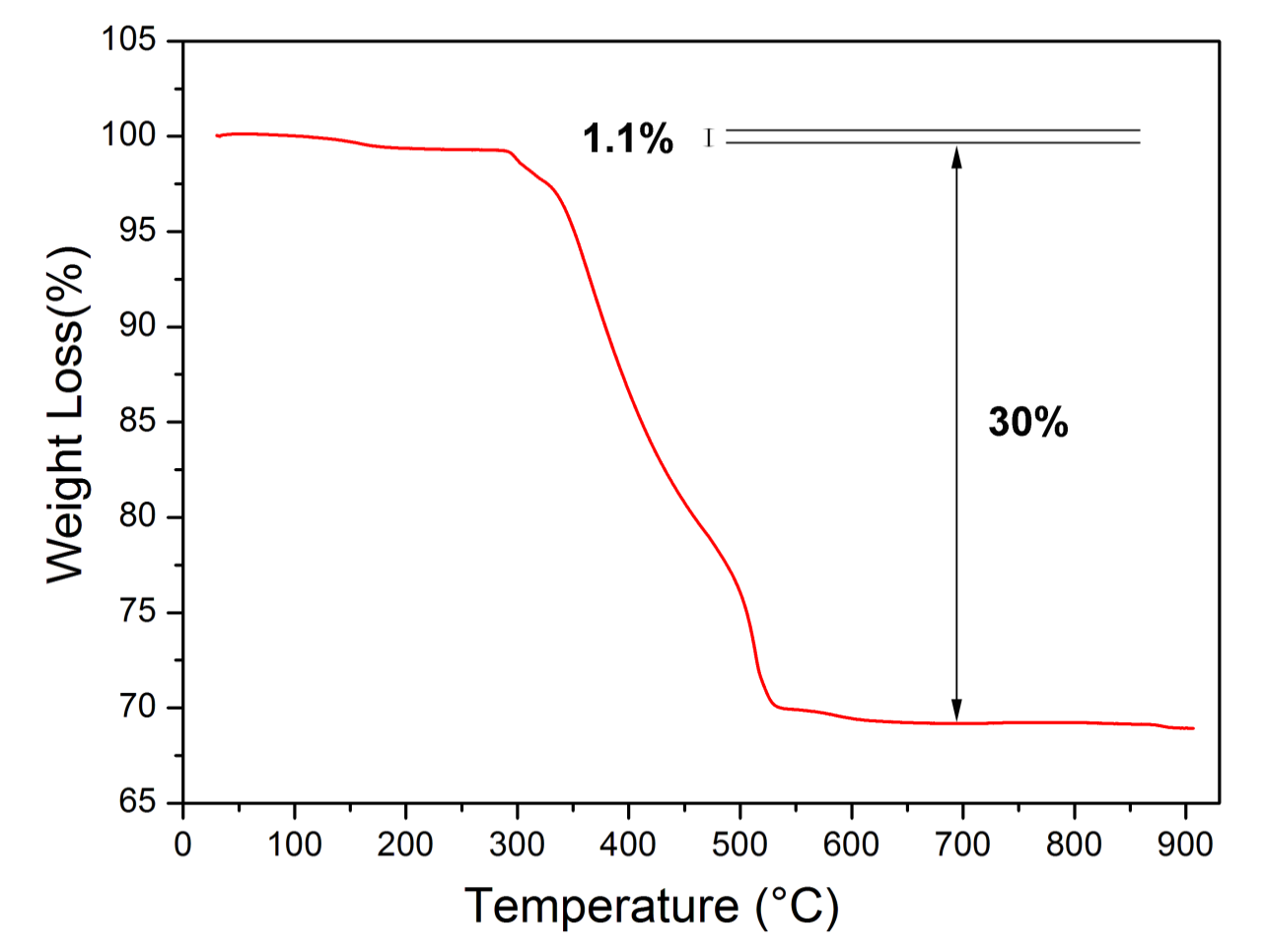


**Figure S2.** Low magnification TEM image of the MO-15, clearly showing the porous structure of BHP-Mn_2_O_3_-SCs.


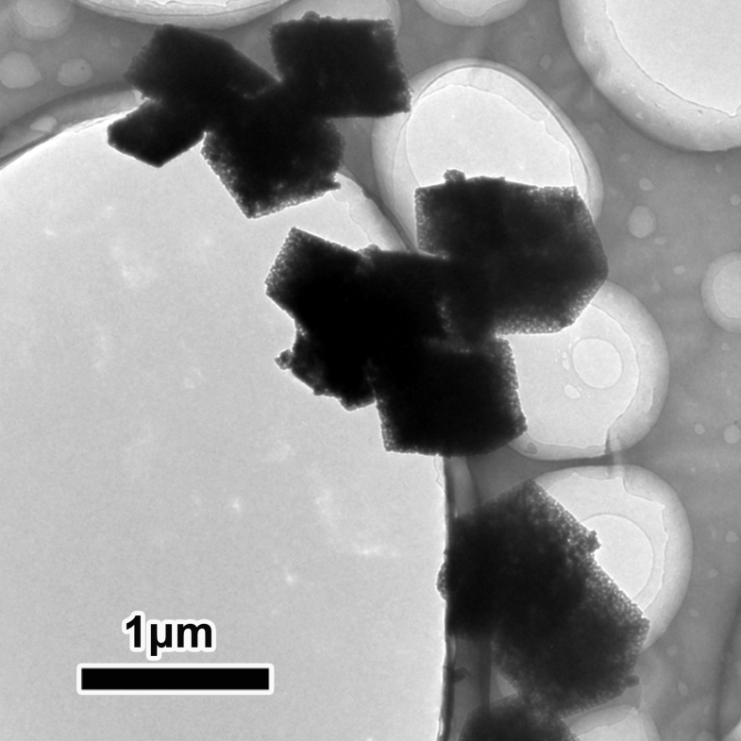


**Figure S3.** High magnification TEM image of the parallelepiped shape of MO-15 shown in Figure. 2c, clearly showing the hierarchically porous structure of the BHP-Mn_2_O_3_-SCs.


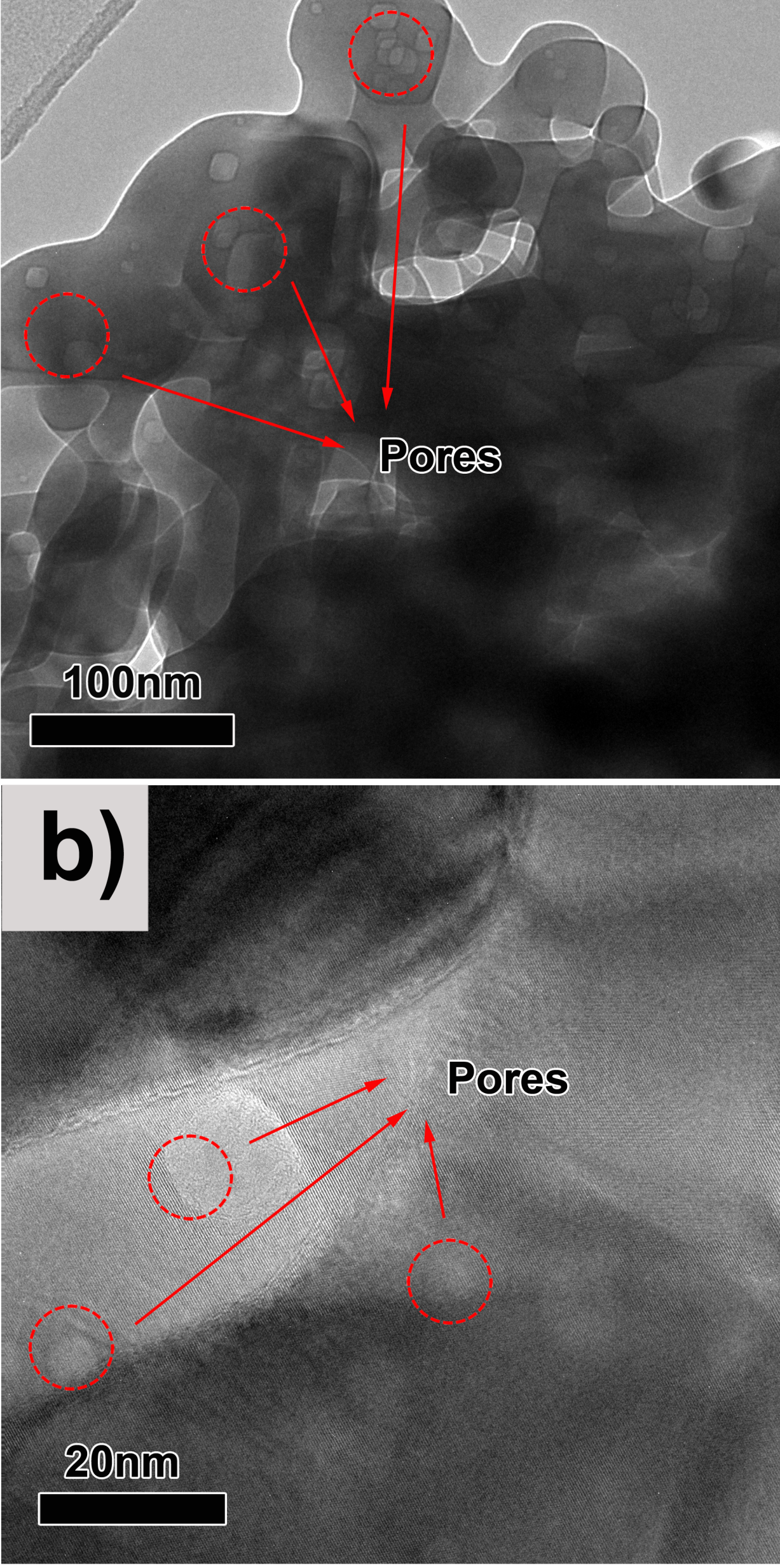


**Figure S4.** SEM image of the MnCO_3_ at the reaction time of 0.5 h, demonstrating the formation of parallelepiped MnCO_3_ at the early growth stage.


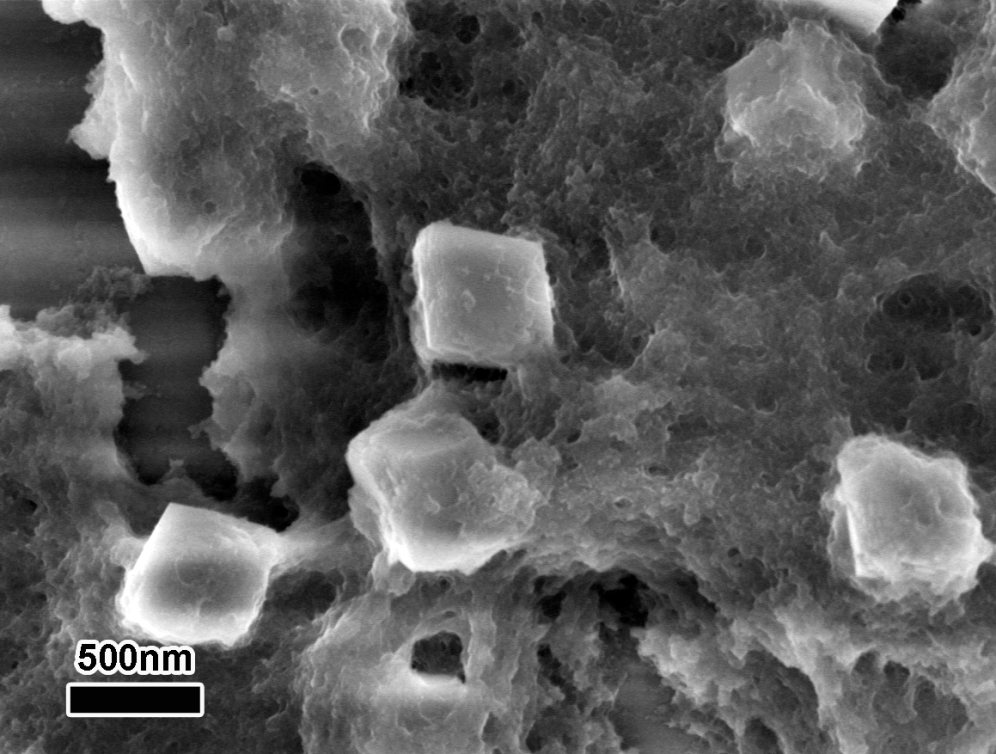


**Figure S5.** The XRD and SEM images of the MnCO_3_ obtained using different amounts of water. a) XRD patterns of all three samples; b) MnCO_3_-5; c) MnCO_3_-15; d) MnCO_3_-30. The XRD patterns show the pure MnCO_3_ with a rhombohedral structure (JCPDS No: 44-1472). The sizes of the three MnCO_3_ samples are ~500 nm, 700 nm and 1.2 μm, respectively.


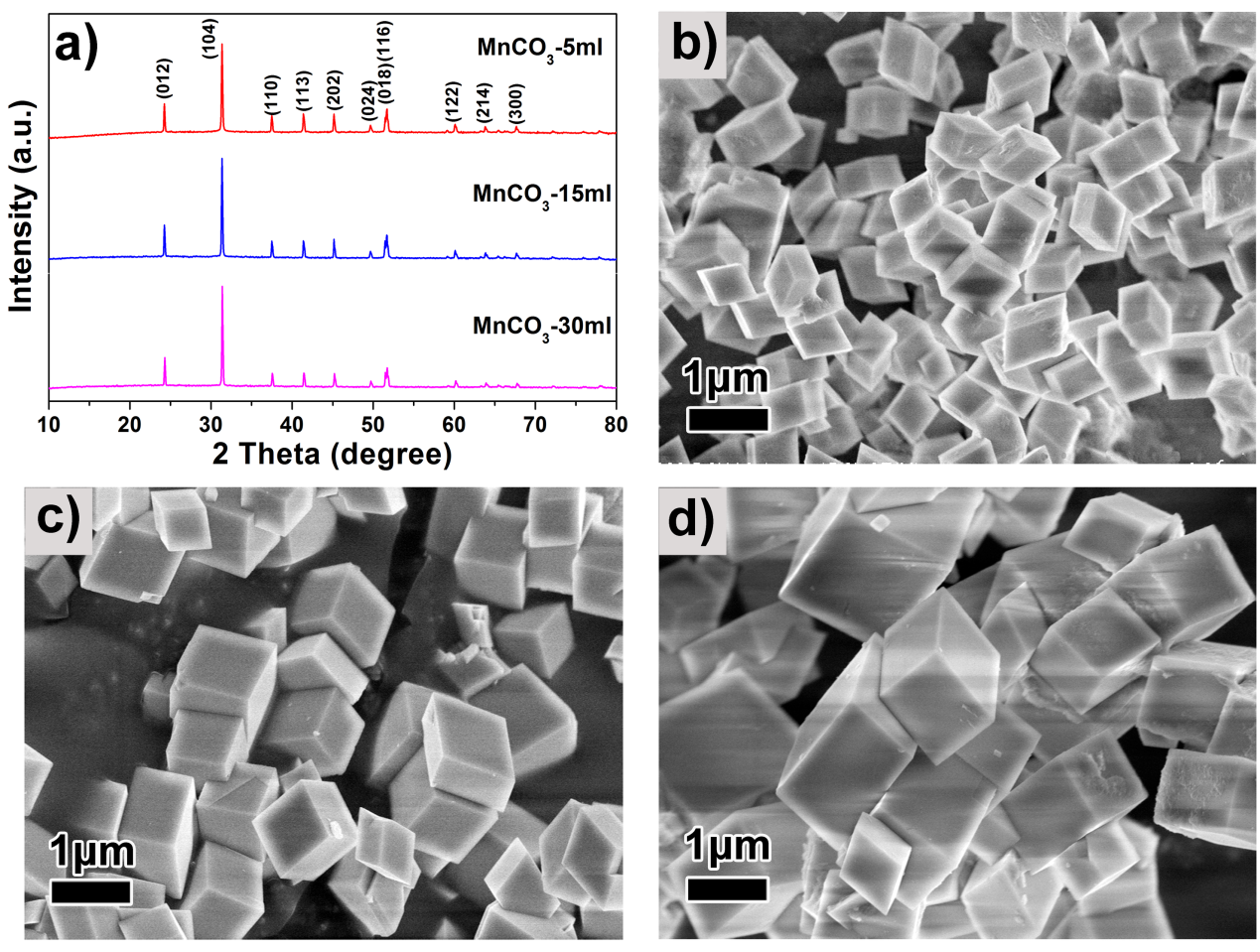


**Figure S6.** N_2_ sorption isotherms of the as-prepared BHP-Mn_2_O_3_-SCs: a) MO-5 and b) MO-30. The insets are their pore size distributions. The BET speciﬁc surface areas were measured to be 21 m^2^ g^-1^ for MO-5 and 28 m^2^ g^-1^ for MO-30 (34 m^2^g^-1^ for MO-15), respectively. From the XRD and SEM results, the lowest BET surface area of MO-5 should be caused by the Mn_5_O_8_ impurity, which blocks part of the pores in the surface. In addition, the BJH analyses of MO-5 and MO-30 (the insets in Figure. S6a and S6b) also show their hierarchically porous structures.


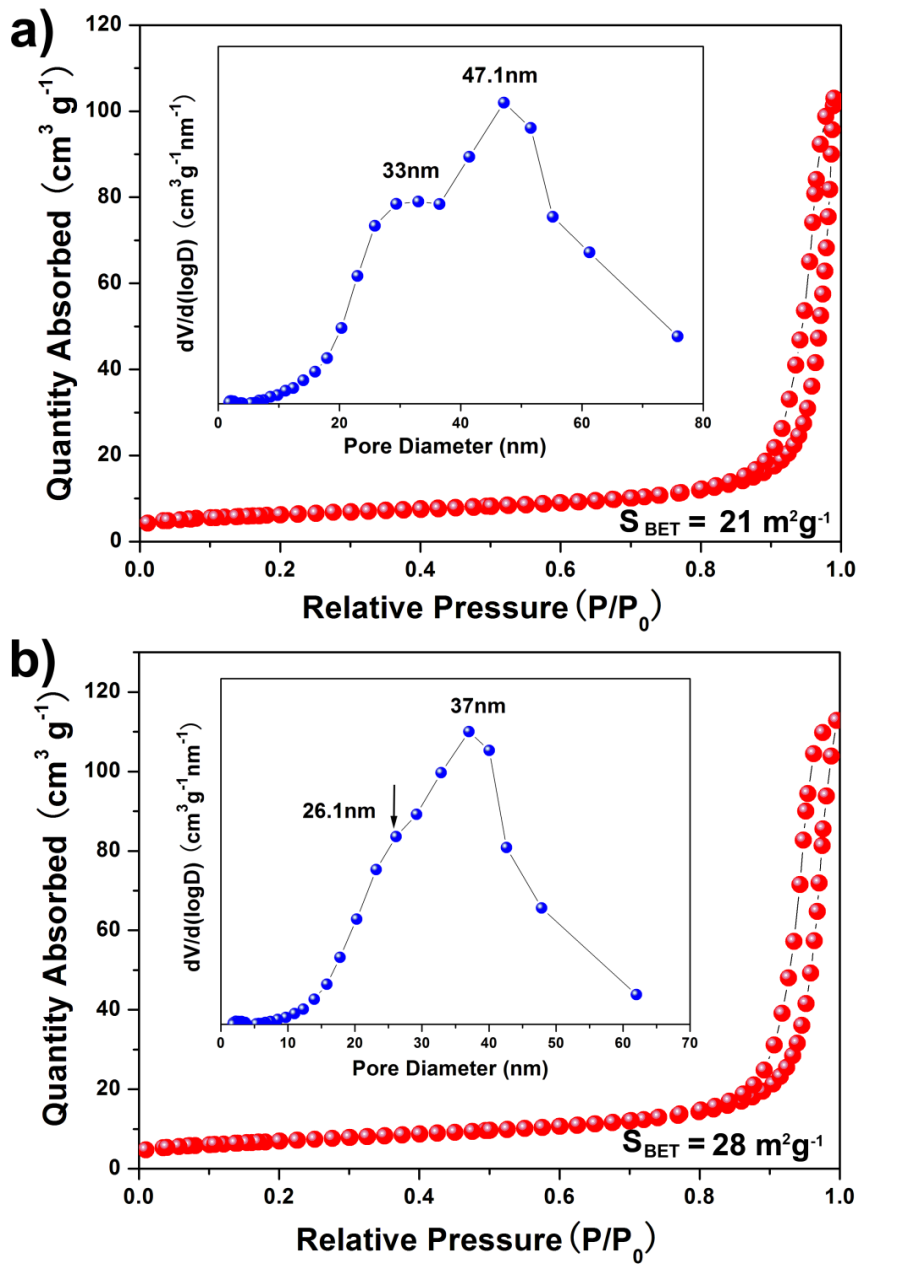


**Figure S7**. The TEM images of the BHP-Mn_2_O_3_-SCs from the MO-30 sample: a) low magnification; b) SAED pattern; c) high magnification; d) HRTEM image. The insets in d) is the crystal structure of Mn_2_O_3_ along the [001] direction.


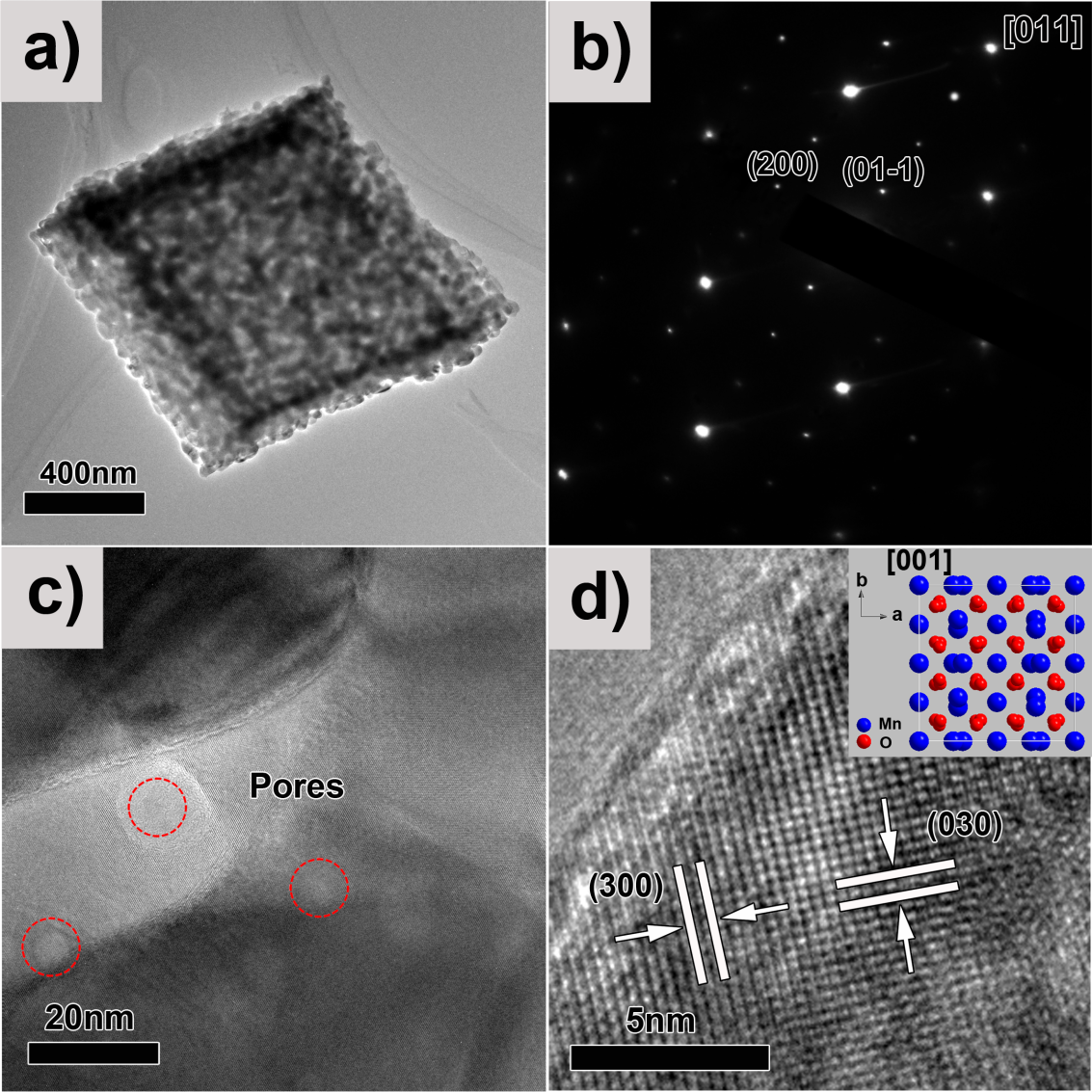


**Figure S8.** Cyclic voltammograms at a scanning rate of 0.1 mV s^-1^ in the voltage range of 0 ~ 3 V versus Li/Li^+^: a) MO-5, b) MO-15, c) MO-30.


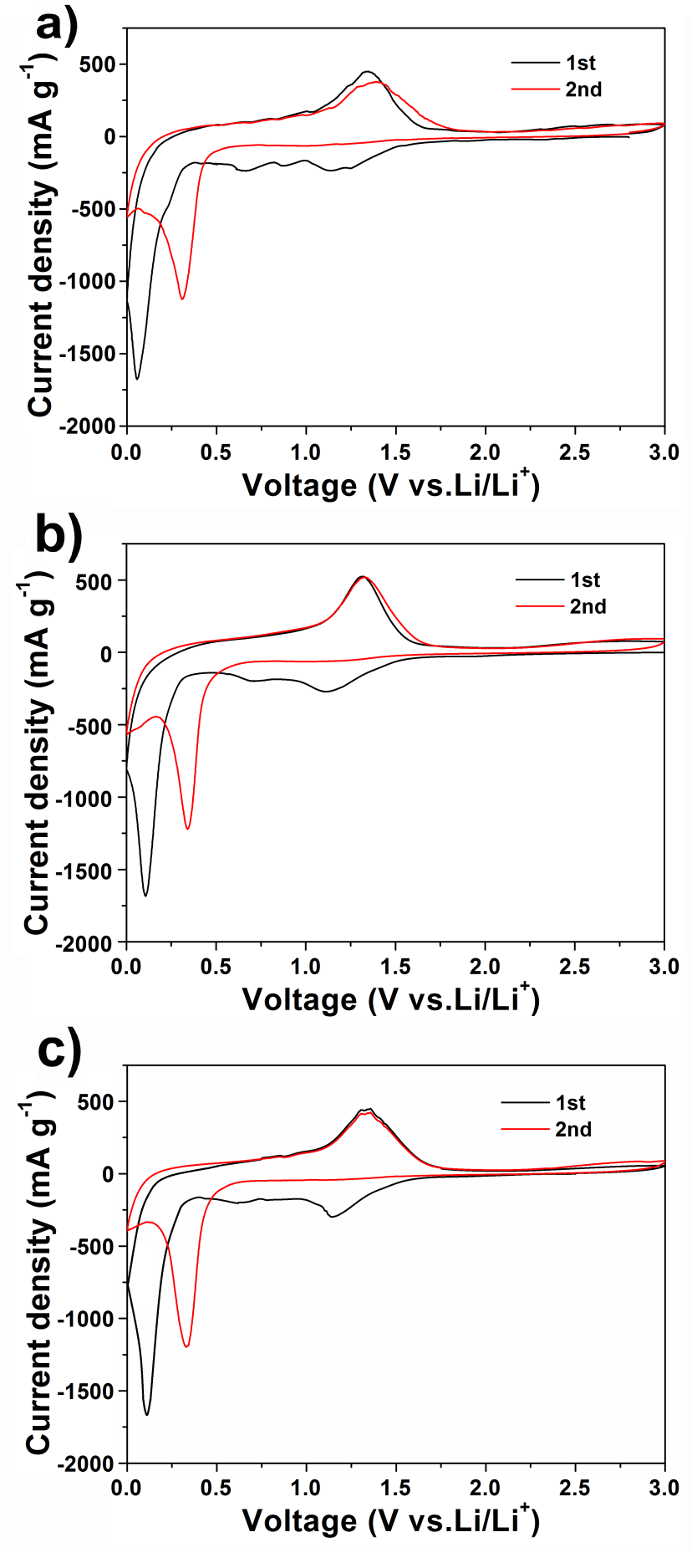


**Figure S9.** The SEM images of the electrodes after 50 discharge-charge cycles at 100 mA g^-1^: a) MO-5; b) MO-15 and c) MO-30. The insets show magnified views of the corresponding samples. The SEM images clearly show three dimensional hierarchically porous single crystal structures remain after charge/discharge cycle.


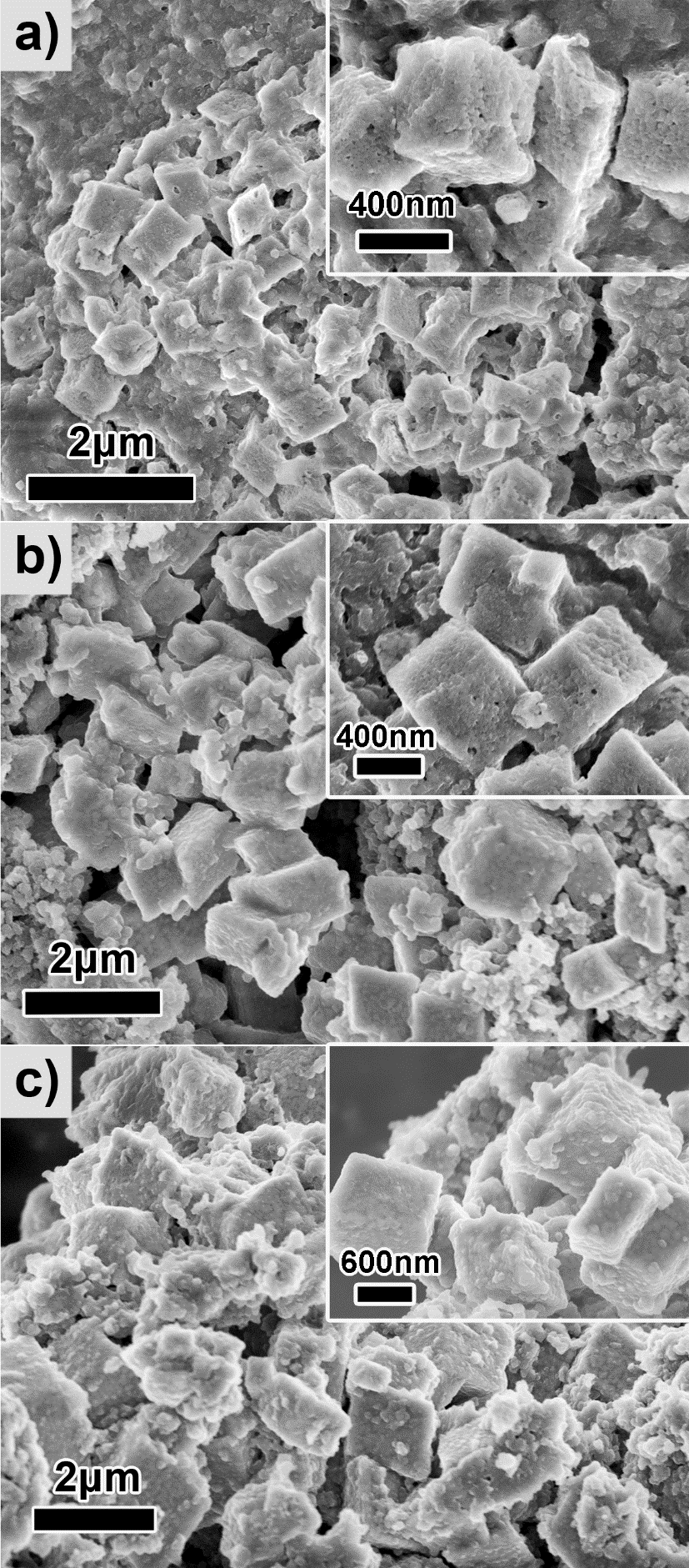


**Figure S10.** The characterizations of solid Mn_2_O_3_ nanoparticles: a) XRD pattern; b) SEM images, showing the Mn_2_O_3_ is composed of nanoparticles and short nanorods; c) cycling performance of BHP-Mn_2_O_3_-SCs and solid Mn_2_O_3_ nanoparticles at 100 mA g^-1^; d) rate capability of BHP-Mn_2_O_3_-SCs and solid Mn_2_O_3_ nanoparticles.


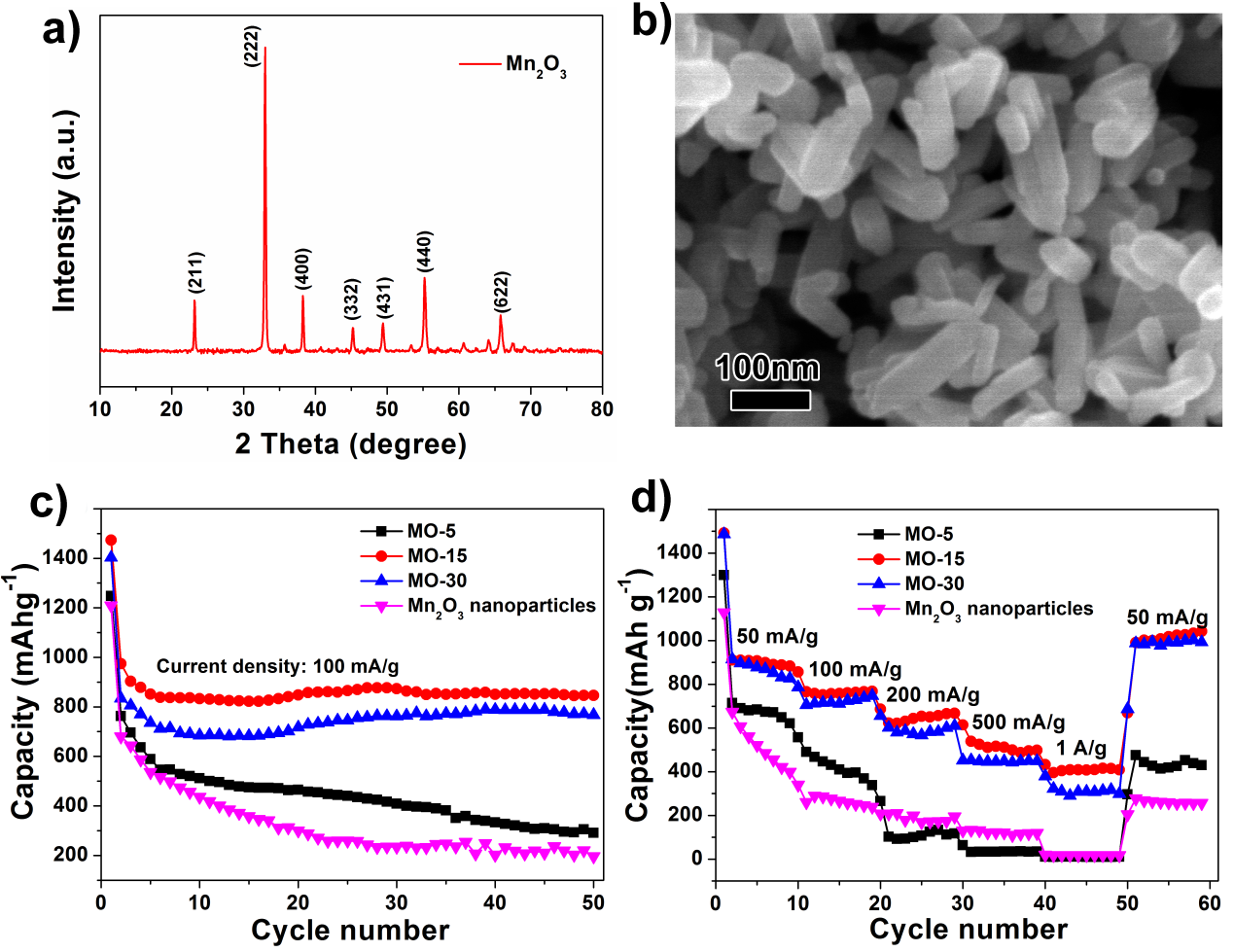

Supplement: Supplementary Information [file srep14686-s1.doc]
